# Nitrogen-Doped Cu_2_O Thin Films for Photovoltaic Applications

**DOI:** 10.3390/ma12183038

**Published:** 2019-09-19

**Authors:** Ørnulf Nordseth, Raj Kumar, Kristin Bergum, Irinela Chilibon, Sean Erik Foss, Edouard Monakhov

**Affiliations:** 1 Institute for Energy Technology (IFE), P.O. Box 40, NO-2027 Kjeller, Norway; Sean.Foss@ife.no; 2 Department of Physics/Center for Materials Science and Nanotechnology (SMN), University of Oslo, P.O. Box 1048, Blindern, NO-0316 Oslo, Norway; raj.kumar@smn.uio.no (R.K.); kristin.bergum@smn.uio.no (K.B.); eduard.monakhov@fys.uio.no (E.M.); 3 National Institute of Research and Development for Optoelectronics (INOE-2000), Bucharest-Magurele, Str. Atomiștilor 409, RO-077125 Măgurele, Romania; chilib@inoe.ro

**Keywords:** cuprous oxide, doping, nitrogen, thin film, magnetron sputtering

## Abstract

Cuprous oxide (Cu_2_O) is a p-type semiconductor with high optical absorption and a direct bandgap of about 2.1 eV, making it an attractive material for photovoltaic applications. For a high-performance photovoltaic device, the formation of low-resistivity contacts on Cu_2_O thin films is a prerequisite, which can be achieved by, for instance, nitrogen doping of Cu_2_O in order to increase the carrier concentration. In this work, nitrogen-doped p-type Cu_2_O thin films were prepared on quartz substrates by magnetron sputter deposition. By adding N_2_ gas during the deposition process, a nitrogen concentration of up to 2.3 × 10^21^ atoms/cm^3^ in the Cu_2_O thin films was achieved, as determined from secondary ion mass spectroscopy measurements. The effect of nitrogen doping on the structural, optical, and electrical properties of the Cu_2_O thin films was investigated. X-ray diffraction measurements suggest a preservation of the Cu_2_O phase for the nitrogen doped thin films, whereas spectrophotometric measurements show that the optical properties were not significantly altered by incorporation of nitrogen into the Cu_2_O matrix. A significant conductivity enhancement was achieved for the nitrogen-doped Cu_2_O thin films, based on Hall effect measurements, i.e., the hole concentration was increased from 4 × 10^15^ to 3 × 10^19^ cm^−3^ and the resistivity was reduced from 190 to 1.9 Ω⋅cm by adding nitrogen to the Cu_2_O thin films.

## 1. Introduction

Cuprous oxide (Cu_2_O) is considered an attractive material for photovoltaic applications since it is a p-type semiconductor with high optical absorption and a direct bandgap of about 2.1 eV, yielding a theoretical power conversion efficiency limit close to 20% under 1 sun illumination [1]. To construct a p-n heterojunction, Cu_2_O can be combined with various n-type metal oxide materials, such as for example ZnO, and accordingly, one can foresee a heterojunction solar cell fully based on low-cost, abundant, and non-toxic metal oxides [2]. The highest power conversion efficiency achieved experimentally for a n-ZnO/p-Cu_2_O heterojunction solar cell is currently 8.1% [3], suggesting that further development of Cu_2_O-based solar cells is required in order to realize their full potential [4]. A possible application of Cu_2_O-based solar cells could be to combine them with conventional crystalline silicon (c-Si) solar cells in a mechanical stack of independently connected cells, enabling low-energy photons to be transmitted through the Cu_2_O-based top subcell for subsequent absorption in the c-Si bottom subcell [5]. Such four-terminal tandem cell configuration has the potential to reach a power conversion efficiency of above 30% under 1 sun illumination [6]. One possible way to enhance the performance of the ZnO/Cu_2_O heterojunction solar cell could be to introduce a highly doped p-type layer at the back side of the Cu_2_O absorber layer to reduce the charge carrier recombination at the rear surface and to form a low contact-resistance interface with low optical absorption [7,8], i.e., introduce a carrier (hole) selective passivating contact layer [9]. A highly doped layer can, for instance, be obtained by tuning of the electrical properties of Cu_2_O by adding foreign atoms, such as nitrogen which is an abundant and non-toxic element that can be straightforwardly incorporated into the Cu_2_O lattice [10]. Ideally, the doping should modify the electrical properties of Cu_2_O without considerably affecting the physical and chemical properties. Previous investigations have suggested that nitrogen is a very effective p-type dopant when mixed into Cu_2_O [11], acting as a substitutional impurity for oxygen atoms [10]. This dopant introduction should not result in formation of the deleterious CuO phase, which is detrimental for the electrical properties of the thin film. Moreover, a previous study on the effects of nitrogen doping on the optical properties of Cu_2_O thin films indicated that the optical band gap could be affected by the incorporation of nitrogen [12].

The objective of this work was to investigate the effect of nitrogen doping on the electrical, structural, and optical properties of p-type Cu_2_O thin films synthesized by direct current (DC) magnetron sputter deposition. The amount of nitrogen incorporated into the deposited Cu_2_O thin films for various amount of N_2_ gas in the process gas mix was analyzed by secondary ion mass spectroscopy (SIMS). Moreover, the crystallographic, morphological, optical, and electrical properties for nitrogen-doped Cu_2_O thin films were determined from X-ray diffraction (XRD), atomic force microscopy (AFM), spectrophotometric, and Hall effect measurements, respectively. We showed that the electrical conductivity can be significantly enhanced by doping the Cu_2_O thin films with nitrogen. For instance, the concentration of holes can be increased by more than three orders of magnitude by adding nitrogen into the Cu_2_O thin films. Also, we showed that the incorporation of nitrogen into the Cu_2_O thin films did not significantly affect surface morphology or the optical properties, and that the nitrogen-doped Cu_2_O thin films remained phase pure.

## 2. Materials and Methods

Cu_2_O thin films were deposited on quartz substrates by reactive sputtering of a Cu target (99.999%) using a DC magnetron sputtering system (Semicore Triaxis, Livermore, CA, USA). 1 × 1 cm^2^ quartz substrates were cleaned in piranha solution (H_2_SO_4_ + H_2_O_2_) and rinsed in deionized water, blown dry with nitrogen, and loaded into the deposition chamber. The base pressure of the chamber was ~7 × 10^−7^ mbar. Prior to deposition, the Cu target was pre-sputtered for 15 min. During deposition, the substrate temperature was kept at 400 °C and the sample stage was rotated at a constant speed of 12 rotations per minute. The deposition power was fixed at 100 W. The nitrogen-doped Cu_2_O (N:Cu_2_O) thin film samples were prepared by varying the flow of process gases as shown in Table 1. The total gas flow was fixed at 50 standard cubic centimeters per minute (sccm) with variation in the Ar/N_2_ flow ratio, whereas the O_2_ flow was fixed at 7.5 sccm for all samples. The deposition time was constant for all samples.

SIMS measurements were carried out to analyze the nitrogen content of the N:Cu_2_O thin films. The nitrogen concentration versus thin film depth was measured using a Cameca IMS 7f micro-analyzer (Gennevilliers, France) with primary beams of 15 keV Cs+ ions. 14N16O molecular ions were detected to quantify the nitrogen content in the thin films. The intensity-concentration calibration was performed using implanted Cu_2_O as a reference sample. Depth conversion of the recorded profiles was performed by measuring the sputtered crater depth using a Dektak 8 stylus profilometer (Bruker, Billerica, MA, USA) and assuming a constant erosion rate. XRD patterns were recorded by a Bruker AXS D8 Discover (Billerica, MA, USA), using Cu Kα-radiation and a Bragg–Brentano configuration. The thin film sample surface was analyzed using a Veeco Innova atomic force microscope (Bruker, Billerica, MA, USA), with a RTESPA Si doped probe. The scanning was carried out in tapping mode and the scanning speed was about 2.5 μm/s. The AFM images had a resolution of 512 × 512 pixels, and SPM Lab Analysis v.7.0 software (Lisboa, Portugal) was used for the image analysis. The UV–vis optical transmittance spectra in the wavelength range from 400 nm to 1500 nm were measured using a Shimadzu SolidSpe-3700 DUV spectrophotometer (Kyoto, Japan), a tungsten light source, and an integrating sphere. Room temperature Hall Effect measurements were carried out to determine the hole mobility, resistivity, and hole carrier density, using a LakeShore 7604 (Westerville, OH, USA) set up with the van der Pauw configuration.

## 3. Results and Discussion

### 3.1. Secondary Ion Mass Spectrometry Analysis

**Figure 1:**
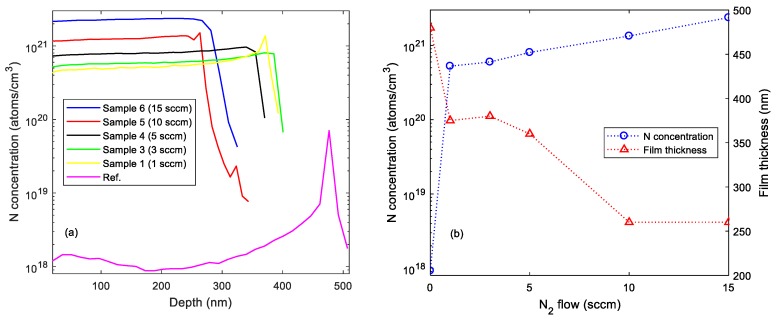
(**a**) Recorded SIMS depth profiles for N:Cu_2_O thin films deposited on quartz. (**b**) Nitrogen concentration and film thickness versus N_2_ gas flow rate.

SIMS depth profiles for the N:Cu_2_O thin film samples are shown in Figure 1a. The measured profiles indicate an increasing nitrogen concentration with increasing N_2_ gas flow rate for the N:Cu_2_O thin film samples. For the Cu_2_O reference sample, a nitrogen concentration around 1 × 10^18^ atoms/cm^3^ was measured in the bulk of the thin film. However, near the interface to the quartz substrate the nitrogen concentration increased to ~7 × 10^19^ atoms/cm^3^ for the Cu_2_O reference sample. This could possibly be a matrix effect due to deviation of the stoichiometry near the interface, or it could be related to ion mass interference, e.g., ^14^N^16^O molecular ions can be interfering with ^30^Si, ^29^SiH, or ^28^SiH_2_ ions. Based on the SIMS depth profiles shown in Figure 1a, the nitrogen concentration at a depth of 200 nm as well as the film thickness for the N:Cu_2_O thin films are plotted in Figure 1b versus the N_2_ gas flow. The data suggests that the nitrogen concentration in the N:Cu_2_O thin films increases with increasing N_2_ gas flow during the deposition process. For example, by increasing the N_2_ gas flow from 1 to 15 sccm, the nitrogen concentration in the N:Cu_2_O thin film is increased from 5.2 × 10^20^ to 2.3 × 10^21^ atoms/cm^3^. Moreover, the film thickness decreases with increasing N_2_ gas flow rate, suggesting that the deposition rate for the N:Cu_2_O thin film decreases with increasing N_2_/Ar gas flow ratio during the sputter deposition process.

### 3.2. Structure and Morphology

**Figure 2:**
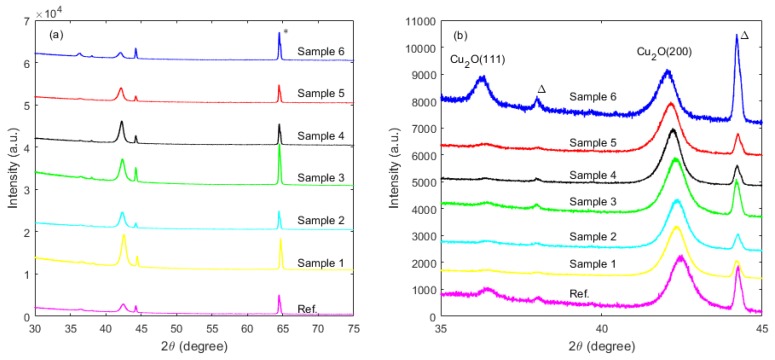
2*θ*–*θ* scans for N:Cu_2_O thin films on quartz in the range (**a**) 30°–75° and (**b**) 35°–45°. The asterisk * indicates the peak of the quartz substrate, whereas Δ indicates the peaks of the sample holder.

Figure 2a shows XRD (*θ*–2*θ*) scans for the N:Cu_2_O thin film samples in the range 30°–75°, whereas the corresponding zoom-in for the range 35°–45° is shown in Figure 2b. The XRD data suggest that the N:Cu_2_O thin films have a polycrystalline structure with diffraction peaks at ~36.6° and ~42.6°, corresponding to reflection from the Cu_2_O(111) and Cu_2_O(200) planes, respectively. The phase of Cu_2_O was determined by comparing the experimental XRD pattern with the Standard Powder Diffraction Cards (ICDD) patterns: 01-071-3645 or space group $O_{h}^{4}$; Pn3m, No. 224 for Cu_2_O [13]. The *θ*–2*θ* scans suggest that there is no obvious structural variation induced by the nitrogen doping and that the films remain phase pure with no presence of other Cu_x_O phases, e.g., there are no diffraction peaks of the cupric oxide (CuO) and copper (III) oxide (Cu_4_O_3_) phases. Also, there are no diffraction peaks related to the Cu_x_N phase observed for the N:Cu_2_O thin film samples in the investigated 2*θ* range between 30° and 75° [14]. Figure 2b shows that the Cu_2_O(111) and Cu_2_O(200) diffraction peaks have shifted towards lower angles, e.g., for Sample 6, the peaks shifted by ~0.4° relative to those for the reference sample, indicating that a small strain has been induced by the nitrogen doping. Furthermore, the Cu_2_O(200) diffraction peak is less dominant with respect to the Cu_2_O(111) diffraction peak for Sample 6 compared to that for the N:Cu_2_O thin film samples with less nitrogen concentration.

**Figure 3:**
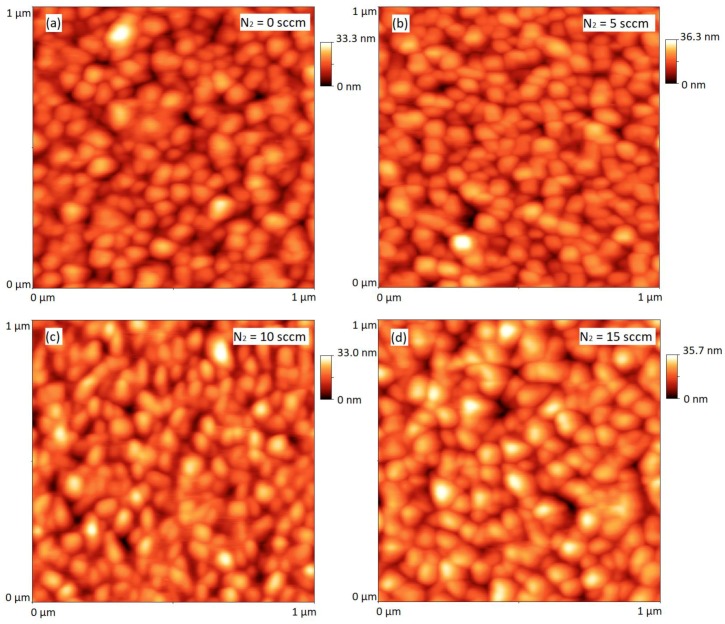
AFM images (1 µm × 1 µm size) for (**a**) the reference sample, (**b**) Sample 4, (**c**) Sample 5 and (**d**) Sample 6.

Figure 3 shows AFM images of three different N:Cu_2_O thin film samples (for 5, 10 and 15 sccm N_2_ gas flow rates), along with the Cu_2_O reference sample. The AFM images suggest that there is no significant change in the surface morphology of the sputter deposited thin films when the N_2_ gas flow rate is increased from 0 to 15 sccm, i.e., the grain size and texture is approximately the same for all four AFM images. The root-mean square surface roughness (*R_RMS_*) for each thin film sample was extracted from the recorded AFM images. *R_RMS_* is defined as the standard deviation of the surface height profile from the mean height, given by:

(1) $$R_{RMS}={\left[\frac{1}{N}\sum_{n-1}^{n}{\left(h_{i}-\langle h\rangle \right)}^{2}\right]}^{\frac{1}{2}}$$

where *N* is the number of data points in the image, *h*_i_ is the height of the *i*th pixel, and *h* is the mean height of the image [15]. In Table 2, the *R_RMS_* is given for each sample analyzed with AFM. The data suggests that *R_RMS_* varies between approximately 3 to 5 nm for all samples, and thus, the increase in N_2_ gas flow rate up to 15 sccm has no major impact on the surface roughness of the N:Cu_2_O thin films.

### 3.3. Optical Properties

**Figure 4:**
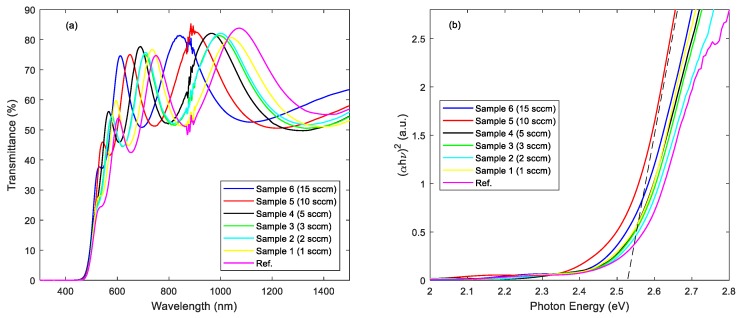
(**a**) Optical transmittance spectra and (**b**) corresponding Tauc plots for N:Cu_2_O thin films and Cu_2_O reference sample on quartz. The optical band gap energy for each sample was estimated from extrapolation to the abscissa (indicated by the dashed line).

The optical transmittance spectra in the wavelength range from 400 to 1500 nm for the N:Cu_2_O thin film samples as well as for the Cu_2_O reference sample are shown in Figure 4a. The data suggest that the thin films exhibit a transmittance of around 70% from the visible to the infrared wavelength range, whereas the high energy photons (λ < 550 nm) are absorbed. Thus, when applied, for instance, as a highly doped p-type N:Cu_2_O layer at the back side of the Cu_2_O absorber layer in a Cu_2_O/c-Si heterojunction tandem cell, the transmission of low-energy photons through the ZnO/Cu_2_O top subcell will not be substantially affected. The optical absorption edges for the N:Cu_2_O thin film samples were estimated by performing a Tauc plot analysis based on the optical transmittance spectra [16]. The resulting (α⋅hν)^1/n^ versus (hν) curves (Tauc plots) for the N:Cu_2_O thin films as well as for the Cu_2_O reference sample are shown in Figure 4b, where α is the optical absorption coefficient and hν is the energy of the incident photons. For n = 1/2, the data show a linear relationship, which indicates a direct allowed optical transition in the N:Cu_2_O thin films as well as for the Cu_2_O reference sample [12]. By extrapolating the hν axis intercept of the line fitted on the linear portion of the Tauc plots, indicated by the dashed line in Figure 4b, the optical band gap energy (E_g_) can be estimated. The Tauc plots suggest that the optical band gap energy is approximately 2.53 ± 0.02 eV for all the analysed films, which is consistent with the values for the optical band gap energy of N:Cu_2_O films reported in the literature [14]. We observe that there is no apparent band-edge shift induced by the nitrogen doping of the Cu_2_O films, which is consistent with the data reported by Ishizuka et al. [10]. However, Nakano et al. observed a possible optical band gap widening for N:Cu_2_O thin films (E_g_ enlarged from 2.1 to 2.5 eV), as a result of a structural change induced by the nitrogen doping [12]. The XRD patterns shown in Figure 2b suggest that the nitrogen doping induces no significant structural change, which could be the reason why a band-edge shift is not observed for the sputter deposited Cu_2_O thin films analyzed in this work.

### 3.4. Electrical Properties

**Figure 5:**
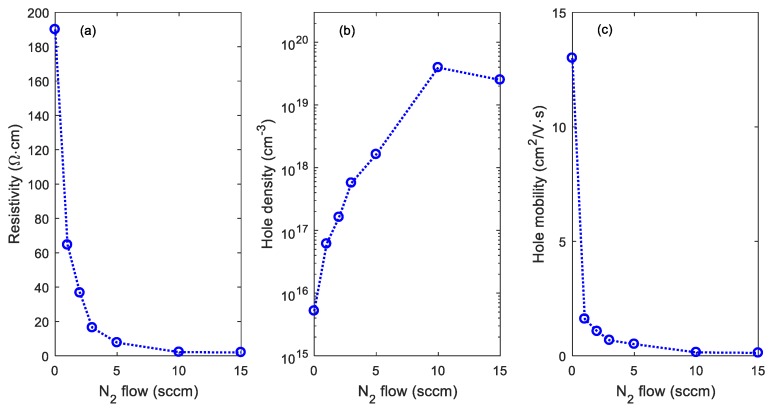
(**a**) Film resistivity, (**b**) majority carrier (hole) density, and (**c**) majority carrier (hole) mobility versus N_2_ gas flow rate for N:Cu_2_O thin films on quartz.

The film resistivity, majority carrier (hole) density, and majority carrier (hole) mobility as a function of N_2_ gas flow rate for the N:Cu_2_O thin films on quartz are presented in Figure 5a–c, respectively. A p-type conductivity is observed for all samples analyzed in this work. Figure 5a shows that the film resistivity decreases with increasing N_2_ gas flow rate. A resistivity of 1.9 Ω⋅cm was obtained for a N_2_ gas flow rate of 15 sccm, which is 100 times lower than the resistivity of the Cu_2_O reference sample. The film resistivity corresponds well with the film resistivity reported by Malerba et al. for Cu_2_O films doped with up to 2.5% nitrogen [17]. Furthermore, the hole density increases with the N_2_ gas flow rate, shown in Figure 5b. A hole density of around 3 × 10^19^ cm^−3^ is obtained for a N_2_ gas flow rate of 10 sccm (Sample 5) and 15 sccm (Sample 6), which is more than three order of magnitudes higher than the hole density measured for the undoped Cu_2_O reference sample. These results indicate that nitrogen doping is very effective in controlling the electrical properties of sputter deposited Cu_2_O thin films. Figure 5c shows that the hole mobility decreases with increasing N_2_ gas flow rate. The hole mobility is reduced from 13 cm^2^/V⋅s for the undoped Cu_2_O reference sample to 0.1 cm^2^/V⋅s for Sample 6, processed with a N_2_ gas flow rate of 15 sccm. The electrical characteristics for these films are in agreement with those reported earlier for sputter-deposited polycrystalline N:Cu_2_O thin films on glass [10,11]. The reduction of the carrier mobility with increasing N content can be ascribed to the presence of nitrogen atoms, acting as impurity scattering centers for free holes [11]. This suggests that the N atoms are in substitutional positions, which forms free holes by introducing shallow acceptor states in the band gap [10].

## 4. Conclusions

In summary, nitrogen-doped Cu_2_O thin films were synthesized by reactive DC magnetron sputtering. Thin film samples were prepared by varying the Ar/N_2_ gas flow ratio, while keeping the O_2_ and the total gas flow rate fixed. SIMS depth profile analysis suggests that the nitrogen concentration in the N:Cu_2_O thin films increases with increasing N_2_ gas flow rate during the sputter deposition process. For example, by increasing the N_2_ gas flow rate from 1 to 15 sccm, the nitrogen concentration in the N:Cu_2_O thin film increases from 5.2 × 10^20^ to 2.3 × 10^21^ atoms/cm^3^. XRD characterization suggests that the N:Cu_2_O films have a polycrystalline structure with a preferred (111) and (200) orientation. There is no obvious structural change induced by the nitrogen doping and the films remain phase pure with no presence of other Cu_x_O or Cu_x_N phases, which can be detrimental for the application of these films in heterojunction devices. The surface morphology of the sputter deposited Cu_2_O thin films is not significantly affected by the nitrogen doping, as determined from AFM images. The *R_RMS_* varies between 3–5 nm for the thin film samples with the N_2_ gas flow rate ranging from 0 to 15 sccm. Tauc plot analysis based on the optical transmittance spectra in the wavelength range from 400 to 1500 nm shows that the optical band gap energy is approximately 2.53 ± 0.02 eV, independent of the nitrogen concentration in the N-doped Cu_2_O thin films, i.e., there is no apparent band-edge shift induced by the nitrogen doping. Room temperature Hall effect measurements show that the N:Cu_2_O thin films display p-type conductivity and that the resistivity decreases with increasing N_2_ gas flow rate. A resistivity of 1.9 Ω⋅cm was obtained for a N_2_ gas flow rate of 15 sccm, compared to a resistivity of 190 Ω⋅cm the undoped Cu_2_O reference sample. The majority carrier (hole) density for the N:Cu_2_O thin films increases with the N_2_ gas flow rate, e.g., a hole density of ~3 × 10^19^ cm^−3^ was obtained for a N_2_ gas flow rate of 10 sccm, more than three order of magnitudes higher than the hole density recorded for the undoped Cu_2_O reference sample. Furthermore, the hole mobility was found to decrease with increasing N_2_ gas flow rate, i.e., for the undoped Cu_2_O reference sample a hole mobility of 13 cm^2^/V⋅s was measured, whereas for the N-doped Cu_2_O thin film sample processed with a N_2_ gas flow rate of 15 sccm a hole mobility of 0.1 cm^2^/V⋅s was measured.

To conclude, we have shown that the electrical properties of Cu_2_O thin films can be modified by nitrogen doping without considerably affecting the structural and optical properties. Potential applications for p-type N:Cu_2_O films include all-oxide based p-n heterojunction devices, such as p-Cu_2_O/n-ZnO photodetectors and solar cells. For example, the N:Cu_2_O films can be incorporated at the back side of the Cu_2_O absorber layer in a ZnO/Cu_2_O heterojunction solar cell to reduce the charge carrier recombination at the rear surface and to form a low-resistivity ohmic contact at the rear interface.

## Figures and Tables

**Table 1:** N:Cu_2_O thin film sample naming and corresponding gas flows used during the sputter deposition process.

Sample Name	N_2_/Ar/O_2_ (sccm)
Reference sample	0/42.5/7.5
1	1/41.5/7.5
2	2/40.5/7.5
3	3/39.5/7.5
4	5/37.5/7.5
5	10/32.5/7.5
6	15/27.5/7.5

**Table 2:** N:Cu_2_O thin film samples and corresponding root-mean square surface roughness (*R_RMS_*) extracted from recorded AFM images.

Sample Name	N_2_ Flow (sccm)	*R_RMS_* (nm)
Reference sample	0	4.40
1	1	3.90
2	2	4.51
3	3	3.13
4	5	4.63
5	10	4.86
6	15	5.45
